# MEGA11: Molecular Evolutionary Genetics Analysis Version 11

**DOI:** 10.1093/molbev/msab120

**Published:** 2021-04-23

**Authors:** Koichiro Tamura, Glen Stecher, Sudhir Kumar

**Affiliations:** 1 Department of Biological Sciences, Tokyo Metropolitan University, Tokyo, Japan; 2 Research Center for Genomics and Bioinformatics, Tokyo Metropolitan University, Tokyo, Japan; 3 Institute for Genomics and Evolutionary Medicine, Temple University, Philadelphia, PA, USA; 4 Department of Biology, Temple University, Philadelphia, PA, USA; 5 Center for Excellence in Genome Medicine and Research, King Abdulaziz University, Jeddah, Saudi Arabia

**Keywords:** software, phylogenetics, timetrees, tip dating, neutrality

## Abstract

The Molecular Evolutionary Genetics Analysis (MEGA) software has matured to contain a large collection of methods and tools of computational molecular evolution. Here, we describe new additions that make MEGA a more comprehensive tool for building timetrees of species, pathogens, and gene families using rapid relaxed-clock methods. Methods for estimating divergence times and confidence intervals are implemented to use probability densities for calibration constraints for node-dating and sequence sampling dates for tip-dating analyses. They are supported by new options for tagging sequences with spatiotemporal sampling information, an expanded interactive *Node Calibrations Editor*, and an extended *Tree Explorer* to display timetrees. Also added is a Bayesian method for estimating neutral evolutionary probabilities of alleles in a species using multispecies sequence alignments and a machine learning method to test for the autocorrelation of evolutionary rates in phylogenies. The computer memory requirements for the maximum likelihood analysis are reduced significantly through reprogramming, and the graphical user interface has been made more responsive and interactive for very big data sets. These enhancements will improve the user experience, quality of results, and the pace of biological discovery. Natively compiled graphical user interface and command-line versions of MEGA11 are available for Microsoft Windows, Linux, and macOS from www.megasoftware.net.

## Introduction

The Molecular Evolutionary Genetics Analysis (MEGA) software has continuously grown to meet the need for sophisticated evolutionary analysis to discover organismal and genome evolutionary patterns and processes. It was first released in 1993 to offer the statistical methods of molecular evolution through an interactive interface on the Microsoft Disk Operating System (MS-DOS) (Kumar et al. 1993). For more than 25 years, MEGA’s scope and usefulness have grown through the addition of new methods, tools, and interfaces, resulting in modern integrated software for comparative sequence analysis (Caspermeyer 2018). Initially, MEGA contained distance-based and maximum parsimony methods for molecular phylogenetic analysis (Kumar et al. 1994). The data acquisition and integration of major approaches for aligning sequences were introduced to expand MEGA's scope (Kumar et al. 2004). Afterward, the maximum likelihood (ML) methods and Bayesian methods were added for molecular evolutionary analyses (Tamura et al. 2011). MEGA now contains methods for selecting the best-fit substitution model(s), estimating evolutionary distances and divergence times, reconstructing phylogenies, predicting ancestral sequences, testing for selection, and diagnosing disease mutations (Caspermeyer 2018).

With every new version, MEGA has evolved to harness technological innovations and personal desktops' computational power. MEGA’s interface evolved from its initial MS-DOS character-based format (Kumar et al. 1993) to a rich graphical user interface (GUI) for Microsoft Windows operating system (Kumar et al. 2001). It was then redesigned to become activity-driven (Tamura et al. 2011), followed by the incorporation of web technologies to ensure a consistent use-and-feel across Microsoft Windows and Linux operating systems (Kumar et al. 2018) and macOS (Stecher et al. 2020). MEGA GUI is now fully cross-platform running natively on Windows, Linux, and macOS.

MEGA’s computational core (MEGA-CC) has undergone extensive refactoring, hardening, and expansion over time. It advanced from 16-bit to 32-bit (Kumar et al. 2001), became multithreaded and incorporated multicore parallelization for various calculations (Tamura et al. 2013), and stepped up to 64-bit architecture (Kumar et al. 2016, 2018). MEGA-CC was released for use as a command-line program to address the growing need for batch processing of many data sets and integration into analysis workflows (Kumar et al. 2012; Stecher et al. 2020). With both 32- and 64-bit versions of MEGA currently available for use on the command-line and GUI, MEGA is now a suite of applications that responds to the variety of computing environments currently used by researchers in molecular evolution and phylogenetics. Here, we present key methodological additions and technical improvements in MEGA that comprise version 11.

## Methodological Additions

### Expansion of Relaxed-Clock Dating Facilities

Rapid relaxed-clock methods for estimating divergence times are becoming popular because they are feasible and efficient for large contemporary sequence alignments (Tao, Tamura, Kumar, et al. 2020). MEGA6 first added methods and tools for constructing evolutionary timetrees by implementing the RelTime method, which does not assume a molecular clock (Tamura et al. 2012, 2013). RelTime is known to perform well and has been used to build timetrees in hundreds of research articles (Tao, Tamura, Kumar, et al. 2020). MEGA11 expands on RelTime dating options by advancing the current implementation and adding new facilities for node-dating and tip-dating needed to build timetrees of pathogens, species, and gene families.

#### Calibrating the Clock Using Probability Densities on Node-Constraints

Bayesian relaxed-clock methods have long allowed the use of statistical probability distributions that capture prior knowledge (or belief) about the true divergence times in clock calibration constraints on one or more nodes in the phylogeny. Judicious use of these probability densities can make divergence times more accurate and precise (Tao, Tamura, Mello, et al. 2020). Researchers can now use such probability densities for node calibrations in RelTime estimation of divergence times and confidence intervals (CIs). MEGA implements the Tao, Tamura, Mello, et al. (2020) approach that estimates CIs by simultaneously accounting for variance introduced by the heterogeneity of evolutionary rate among lineages, estimation of sequence divergence using substitution models, and probability densities for node-calibration constraints. This method produces CIs that contain correct times with a high probability, making them much more suitable for biological hypothesis testing than other rapid methods (Tao, Tamura, Kumar, et al. 2020; Tao, Tamura, Mello, et al. 2020). 

For RelTime analyses in MEGA11, ML and distance-based approaches can be used to build a timetree for a given phylogeny and multiple sequence alignment. One may also use only a phylogeny with branch lengths, which extends the usefulness of relaxed-clock methods for phylogenies inferred from nonmolecular data or statistical methodologies not available in MEGA. When a phylogeny with branch lengths is used, the CIs will be narrower because the variance associated with branch length estimation cannot be generated without the original data set used to produce the phylogeny and branch lengths. Nevertheless, these CIs will incorporate variance introduced due to rate variation among lineages and clock calibrations’ uncertainty.

**Fig. 1.**
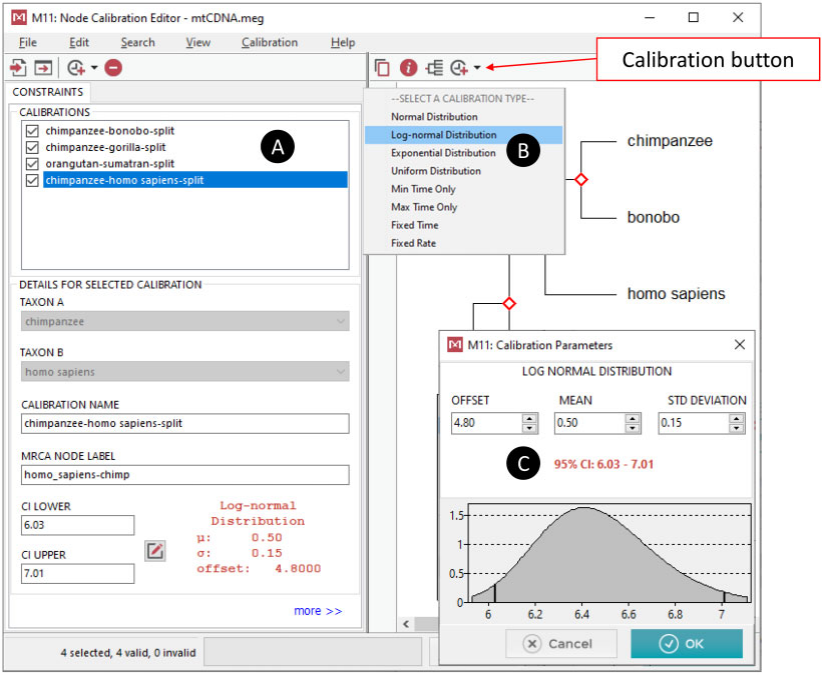
Calibration points for MEGA’s RelTime method are chosen in the *Node Calibration Editor* window (*A*), accessed via the *Timetree Wizard* system (see fig. 2*A*). The *Node Calibration Editor* displays the phylogeny where individual node calibrations and probability densities can be chosen by clicking the calibration button on the top toolbar for the selected node. A dropdown menu (*B*) with several calibration density types is displayed. The *Node Calibration Editor* then prompts the user for required distribution parameters, depending on the distribution selected: normal distribution (mean and standard deviation), lognormal (offset, mean and standard deviation), exponential (offset and decay parameter), uniform (min and max) (*C*).

A calibration density selector has been added to the *Node Calibration Editor* that provides an option to select normal, lognormal, uniform, or exponential density (fig. 1). The user can also specify a minimum or a maximum time bound on a node. The calibration text file format has been extended to specify density information and use calibration densities in MEGA-CC. The *Node Calibration Editor* also includes new functionality to specify a fixed evolutionary rate or a known node time to calibrate the molecular clock. Such assumptions are often used by investigators when independent calibration information is unknown (Hipsley and Müller 2014; Tao, Tamura, Kumar, et al. 2020).

#### Tip-Dating for Sequences with Sampling Times

MEGA now implements a method to estimate timetrees using sampling dates for molecular sequences. They are often used to infer the origin and diversification of pathogens that generally evolve fast enough to track the evolutionary change over months and years (Tao, Tamura, Kumar, et al. 2020). Tip-dating methods are also useful for analyzing ancient molecular sequences. MEGA implements a rapid tip-dating method, RelTime with Dated Tips (RTDT), that produces divergence times and CIs (Miura et al. 2018). One may use ML or distance-based approaches for a given phylogeny and multiple sequence alignment for tip-dating, or a phylogeny with branch lengths and tip dates can be given as the input.

**Fig. 2.**
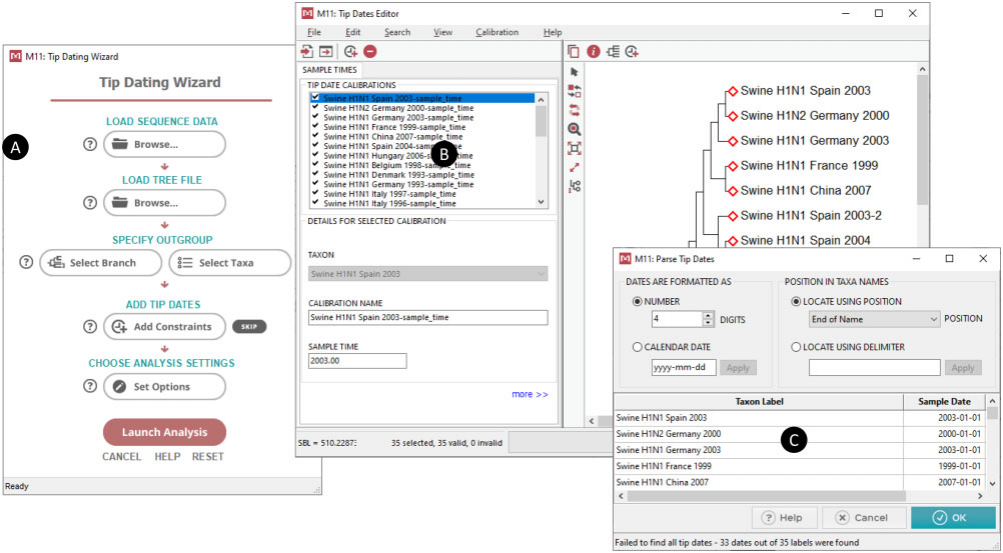
The *Tip Dating Wizard* (*A*) guides the user through the steps required to set up the RTDT analysis. Once a sequence alignment and/or a tree is provided, the user is prompted to specify the outgroup by selecting a node in the *Tree Explorer* or specifying outgroup taxa by name (not shown). Next, sample times are specified using the *Tip Dates Editor* (*B*) with facilities for parsing tip dates (*C*) encoded in taxa names, importing tip dates from a text file, and manually entering the dates. In the next step, the *Analysis Preferences* dialog (not shown) is displayed, allowing the user to set analysis options to estimate branch lengths used by RTDT. The estimated timetree is displayed in the *Tree Explorer* (see fig. 3).

**Fig. 3.**
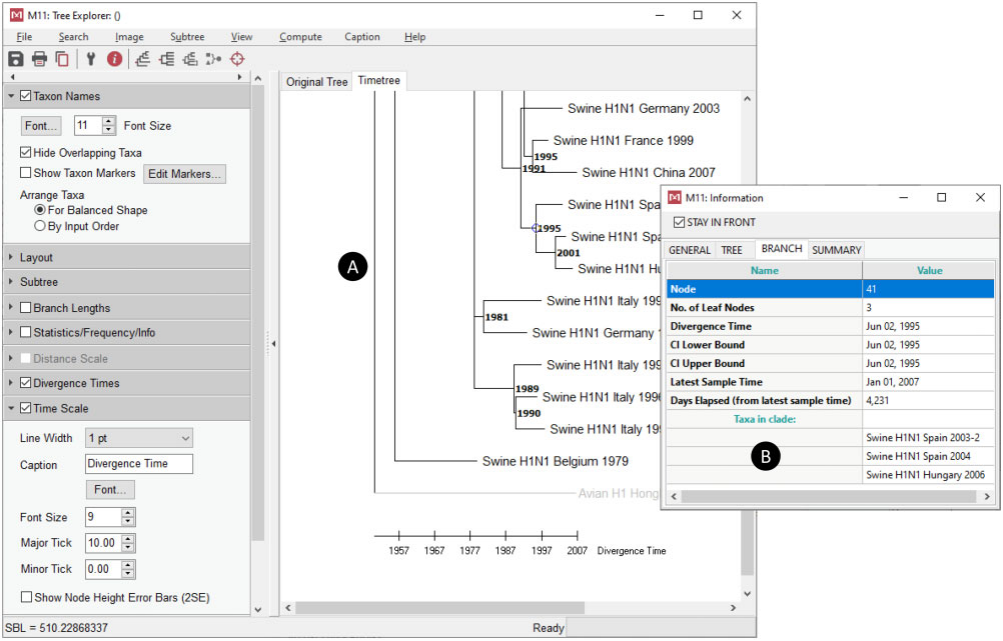
MEGA’s *Tree Explorer* (*A*) is a feature-rich, versatile viewer of phylogenies that provides many interactive exploration and customization facilities. In MEGA11, the new side toolbar of *Tree Explorer* makes formatting, rearrangement, and tree exploration tools more accessible and intuitive. Instead of a thin toolbar with nameless buttons, we have opted for a wide toolbar with text labels identifying each tool. The toolbar can be moved to either side of the window, and it can be toggled in and out of view. To organize related tools by groups and accommodate limited vertical space, collapsible panels are used. With the new toolbar, formatting tools previously displayed in external dialogs are readily accessible, and formats are applied instantly instead of after the user closes the external dialog. In addition to the updated toolbar, there are now options for auto-collapsing of nodes containing clusters of taxa belonging to the same group, user-specified cluster size, or by the branch length difference. For very large trees with many similar sequences, this feature can greatly facilitate the visualization of evolutionary events at a glance. An option has been added to export pairwise patristic distances between taxa to a text file for phylogenies and timetrees. For maximum likelihood and maximum parsimony trees where ancestral sequences are present, an option has been added to navigate through sites where a change in the estimated ancestral state differs between the parent and child on the currently selected branch. The tree information box (*B*) has been updated for timetrees to show branch- and node-specific information, such as earliest and latest sample times in the currently selected subtree, days elapsed between the divergence time for a selected node and the latest sample time, the nearest and furthest tip from a selected node, clade size and clade taxa, and spatiotemporal information if available.

An enhanced *Timetree Wizard* system (fig. 2) walks the user through many steps needed to configure tip-dating analyses, such as loading sequence and tree files, specifying the outgroups, adding sequence sample times, and selecting the analysis options. Sequence sampling times can be specified in multiple ways. MEGA will automatically extract them on-demand when they are included in the sequence name. Spatiotemporal information can also be presented in the input alignment files as meta tags (see description below) or loaded using specially formatted calibration text files. Once computed, the timetree is displayed in the *Tree Explorer* that has been extensively revamped and updated (fig. 3). It now has many more formatting tools, including exporting the timetree, individual divergence times, and CI estimates in a tabular format.

### Detecting Autocorrelation of Evolutionary Rates

MEGA now contains a facility for detecting autocorrelation of evolutionary rates among branches, which is important for understanding molecular evolution patterns and useful as a clock rate prior in Bayesian relaxed-clock analyses. MEGA implements the CorrTest method developed using machine learning, which is accurate and computationally efficient (Tao et al. 2019). The CorrTest implementation in MEGA requires a phylogeny with sequence alignment (or branch lengths) and is accessed through an easy-to-use wizard. This test's final output is a CorrScore between 0 and 1 and a *P*-value, where a high CorrScore and low *P*-value indicates that branch rates among lineages are likely correlated.

### Calculating Neutral Evolutionary Probabilities

According to the neutral theory of molecular evolution, most differences in molecular sequences across species are expected to have little to no impact on fitness (Kimura 1983). Therefore, multispecies sequence alignments have been used to estimate neutral evolutionary probabilities (*EP*) of observing alternative alleles (amino acid residues or nucleotides) in a species, contingent on the given species timetree (Liu et al. 2016). MEGA implements an advanced option for this Bayesian approach in which the species timetree containing relative times is computed automatically by using RelTime (Patel and Kumar 2019). Alleles with *EP* less than 0.05 are nonneutral, whereas evolutionary permissible (neutral) alleles show much higher EPs. Disease-associated amino acid variants in human populations have *EP* < 0.05 and are rarely found in the population (Liu et al. 2016). Many human adaptive variants in populations also have low EPs, that is, nonneutral from an evolutionary perspective, but they show high allele frequencies (Patel et al. 2018). Therefore, one may use *EP*s to diagnose disease mutations and detect candidate adaptive variants. An *EP wizard* system walks the user through the steps required to set up the analysis. The first sequence in the alignment is used automatically as the focal taxon of interest (one can rearrange sequences in the *Sequence Data Explorer*). *EP* values for all possible bases (4 for nucleotides and 20 for amino acids) at each position in the input sequence alignment are reported in a spreadsheet or text format.

## Technological Advances

Although some new user interface elements have already been mentioned above (figs. 1–3), additional technical advances in MEGA11 are as follows.

### Expanded Group Designations

MEGA has long supported a “group” tag for sequences and other operational taxonomic units (OTUs). Using the sequence “group” tags, MEGA offered a group-wise exploration of input data, selection of data subsets, and computational analyses (Kumar 2001). Support for two new tags (“population” and “species”) was added in MEGA7, with the species tags used to mark duplicate genes in multigene family phylogenies (Kumar et al. 2016). In MEGA11, sequences can now be tagged to provide information on the continent, country, city, year, month, day, and time. This spatiotemporal information can be used in tip-dating analyses.

In MEGA11, we have made a MEGA-wide change to use any meta tag to define groups. For example, if one selects the “Year” meta tag for use as a group, they could estimate average diversity within and between sequences sampled in different years (*Distance* menu). In the *Sequence Data Explorer*, one can select/unselect sequences of certain years for phylogenetic analyses. Also, the display of years would be automatically enabled in the *Tree Explorer*, and the feature to collapse sequence clusters will be done by years. Additionally, sequences can be sorted based on years in all the input data and result explorer displays. Therefore, a dynamic designation of groups based on the desired meta tag will enable data exploration and analysis more efficiently.

### Memory Efficient ML Analyses

ML methods are widely used for phylogenetic inference but place high demands on computer memory, becoming increasingly burdensome for bigger sequence alignments analyzed these days. In MEGA11, we have now completed a long-overdue refactoring of ML calculations by adding a step to identify common site configurations, that is, sites where all sequences have the same bases as at some other sites, to utilize computer memory more efficiently. The memory requirements of Maximum Likelihood and Maximum parsimony analysis are reduced (approximately) by the factor of *m*/*L* when there are *m* distinct site configurations in a sequence alignment containing *L* sites. The memory saving can be substantial for multigene and genome-scale alignments. For example, the memory saving was 660 MB (209 vs. 870 MB) for a sequence alignment of 229 birds with 2,728 sites (Claramunt and Cracraft 2015) and 4.5 GB (2.3 vs. 6.8 GB) for an alignment of 162 mammals with 11,010 sites (Meredith et al. 2011). This memory saving does not have any detrimental impact on phylogenetic estimates and computational times because identical site configurations have the same likelihood value. The total log-likelihood is simply the sum of site-configuration log-likelihoods weighted by their frequencies. However, this upgrade required refactoring many different parts of MEGA’s calculation engine, including functions for phylogeny construction and model selection.

### Enhanced GUI for Exploring Large Data Sets

Using a large multiple sequence alignment containing 68,000 genomes and 30,000 bases each, we assessed MEGA GUI's responsiveness during input data file reading, execution of functions in the *Sequence Data Explorer*, estimation of pairwise distances, and building of distance-based phylogenies. We found the GUI to become intermittently unresponsive for such large data sets, which are now common due to resequencing and population sequencing efforts. Consequently, we have moved all potentially long-running operations out of the main GUI thread to background threads in a major overhaul of the source code. Now, large input data files are read rapidly, and calculations of pairwise distance matrices, selection tests, and phylogeny construction for distance-based methods are performed in a background thread. The *Sequence Data Explorer* has been reprogrammed to enable more efficient highlighting of variable sites, and navigation of the sequence alignment has been improved. Also added are options to automatically label sites based on attributes, which annotates sites by providing a one-character label and then using desired labeled sites to subset data for any molecular phylogenetic analysis desired.

## Conclusions

Version 11 of MEGA adds many methods and tools to keep pace with researchers' growing needs. The addition of evolutionary dating methods in MEGA make it easier to estimate species and strain divergence times by using more informative node calibrations and sampling times. The new CorrTest and EP calculations will enable a more robust evaluation of assumptions about biological characteristics of molecular data. The reduction in memory needs of ML-based computations will allow users to analyze much larger data sets than before. The refactoring of distance-based methods’ calculation to run in threads independent of the main graphical interface and other GUI enhancements greatly improve MEGA usability for very large data sets.
